# An Assessment of Carotid Flow Time Using a Portable Handheld Ultrasound Device: The Ideal Tool for Guiding Intraoperative Fluid Management?

**DOI:** 10.3390/mi14030510

**Published:** 2023-02-22

**Authors:** Lauren E. Gibson, James E. Mitchell, Edward A. Bittner, Marvin G. Chang

**Affiliations:** Department of Anesthesia, Critical Care, and Pain Medicine, Massachusetts General Hospital, Boston, MA 02114, USA

**Keywords:** portable ultrasound, point-of-care ultrasound (POCUS), portable point-of-care ultrasound (PPOCUS), volume resuscitation, volume responsiveness, fluid resuscitation, fluid responsiveness, carotid ultrasound, carotid flow time, corrected carotid flow time (ccFT)

## Abstract

Volume resuscitation is a cornerstone of modern anesthesia care. Finding the right balance to avoid inadequate or excess volume administration is often difficult to clinically discern and can lead to negative consequences. Pulse pressure variation is often intraoperatively used to guide volume resuscitation; however, this requires an invasive arterial line and is generally only applicable to patients who are mechanically ventilated. Unfortunately, without a pulmonary artery catheter or another costly noninvasive device, performing serial measurements of cardiac output is challenging, time-consuming, and often impractical. Furthermore, noninvasive measures such as LVOT VTI require significant technical expertise as well as access to the chest, which may not be practical during and after surgery. Other noninvasive techniques such as bioreactance and esophageal Doppler require the use of costly single-use sensors. Here, we present a case report on the use of corrected carotid flow time (ccFT) from a portable, handheld ultrasound device as a practical, noninvasive, and technically straightforward method to assess fluid responsiveness in the perioperative period, as well as the inpatient and outpatient settings.

## 1. Introduction

Volume resuscitation is a cornerstone of modern anesthesia care. Finding the right balance to avoid inadequate or excess volume administration is often difficult to clinically discern and can lead to negative consequences. These consequences range from acute kidney injuries, inadequate cellular perfusion, to pulmonary edema or even frank anasarca leading to poor wound healing and increased hospital length of stay [1,2]. There are many validated tools already at a clinician’s disposal; however, many of these are not well-suited for the operating room given the limitations in space, equipment, and time. For both spontaneously breathing and mechanically ventilated patients, fluid responsiveness can be demonstrated by a significant increase in stroke volume or cardiac output with either a fluid challenge or passive leg raise, the latter of which is not ideal for routine intraoperative use. Pulse pressure variation is often intraoperatively used to guide volume resuscitation; however, this requires an invasive arterial line and is generally only applicable in patients who are mechanically ventilated. Unfortunately, without a pulmonary artery catheter or other costly noninvasive device, performing serial measurements of cardiac output is challenging, time-consuming, and often impractical. Furthermore, LVOT variation to assess for fluid responsiveness may be impractical since it requires significant technical expertise, and there is often limited access to the patient’s chest to obtain these measurements. Other noninvasive techniques such as bioreactance and esophageal Doppler require the use of costly single-use sensors.

ccFT has been validated as a technique for assessing cardiac output [3], where changes in the duration of systolic flow in the carotid artery have been demonstrated to correlate with volume status and are unaffected by respiration, thus reliable in both mechanically ventilated and spontaneously breathing patients [4]. Here, we present a case report on the use of corrected carotid flow time (ccFT) using a portable, handheld ultrasound device to assess fluid responsiveness.

## 2. Case Report

**Figure 1:**
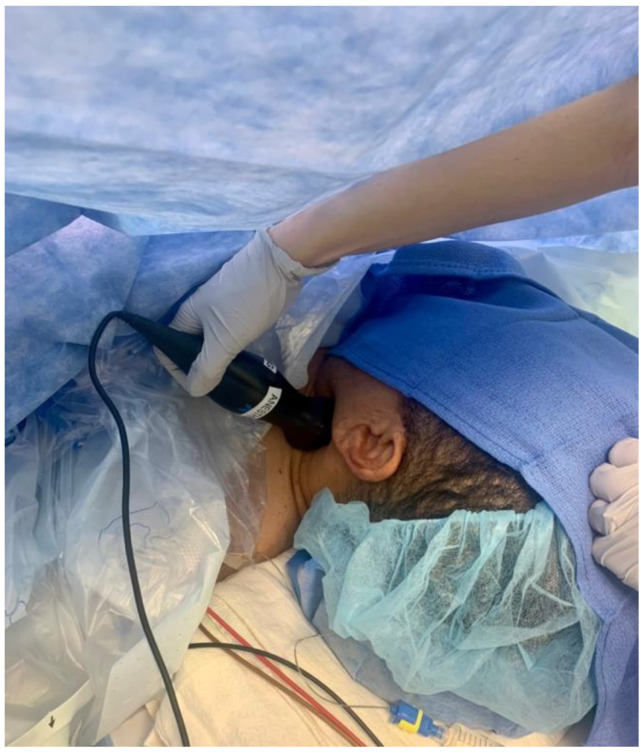
Intraoperative assessment of carotid blood flow performed using a portable handheld ultrasound device (Butterfly iQ) at a patient’s left carotid artery. The handheld device is placed on the patient’s neck, and the carotid artery is identified in transverse section. The device is then rotated 90 degrees to longitudinally image the vessel and align the spectral Doppler in the direction of blood flow. Either the left or right carotid artery can be used, and serial measurements should utilize the same vessel.

Our patient was a 14-year-old girl who was otherwise healthy presenting to the operating room for open reduction and internal fixation of an ankle fracture. She had been NPO for 18 h prior to the start of the surgery. She was 160 cm tall and weighed 65 kg. Her initial heart rate was 114 beats/minute, and her initial blood pressure was 114/65 mmHg. General anesthesia was induced with 200 mg of propofol and 50 mcg of fentanyl, a laryngeal mask airway was inserted, and she was maintained on a concentration of 3.2% inspired sevoflurane throughout the procedure. Carotid flow time was measured 45 min following the induction of anesthesia by placing a handheld ultrasound probe, Butterfly IQ (Butterfly Network, Inc., Guilford, CT, USA), on the patient’s neck (Figure 1) and locating the common carotid artery in the long axis. A pulsed-wave spectral Doppler tracing was then obtained in the center of the artery. The carotid flow time was determined as the duration from the beginning of the systolic waveform to the dicrotic notch and corrected for heart rate using Wodey’s formula [5] to obtain the ccFT in milliseconds (ms): Corrected carotid flow time (ccFT) = carotid flow time + 1.29 (heart rate—60). Using this formula, an increase in ccFT of at least 7 ms after a fluid bolus has been shown to detect fluid responsiveness with good sensitivity [6]. Carotid flow time was measured before and after a 250 cc bolus of lactated Ringer’s. The fluid responsiveness in the same patient was assessed via pulse pressure variation, as well as LVOT VTI variation and ccFT using a traditional ultrasound machine in response to a passive leg raise.

## 3. Results

**Figure 2:**
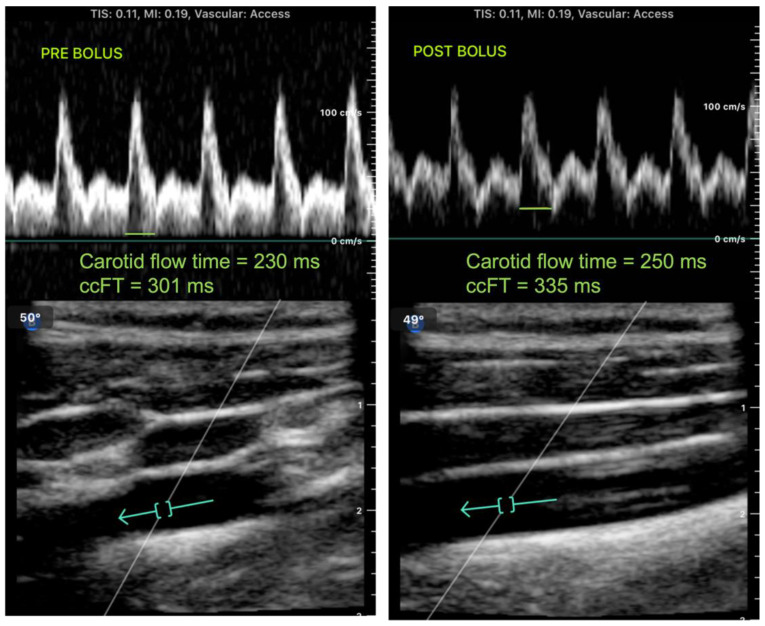
Spectral Doppler tracing (**top**) showing the measurement of carotid flow time intraoperatively on a patient using portable handheld ultrasound before (**left panel**) and after (**right panel**) a 250 cc fluid bolus. The arrows (**bottom**) show the measurement location of the Doppler tracing. The systolic flow time at the common carotid artery was corrected for heart rate based on Wodey’s formula to determine a corrected carotid flow time (ccFT) of 301 ms before a fluid bolus and 335 ms after a fluid bolus, suggesting that this patient is fluid-responsive and may benefit from continued fluid administration.

Figure 2 shows spectral Doppler tracing showing the measurement of carotid flow time intraoperatively on a patient using portable handheld ultrasound before (left panel) and after (right panel) a 250 cc fluid bolus of lactated Ringer’s. The ccFT was found to increase from 301 ms to 335 ms following the 250 cc fluid bolus (3.8 cc/kg), which suggested that the patient was volume-responsive. Her heart rate and blood pressure were 99 beats/minute and 106/51 mmHg, respectively, prior to the fluid bolus, and they remained stable and unchanged immediately following. The same patient was found to be volume-responsive by pulse pressure variation (>12%) as well as LVOT VTI (>12% change) using a traditional ultrasound machine in response to a passive leg raise. She continued to receive an additional 500 cc of lactated Ringer’s solution for a total of 750 cc of fluid administered during the case. She remained hemodynamically stable and normotensive during the case and throughout her post-operative recovery.

## 4. Conclusions

This is the first presentation of the use of a portable, handheld ultrasound device to measure changes in ccFT as a practical, noninvasive, and technically straightforward method to assess fluid responsiveness. Although we demonstrate the calculation of ccFT before and after a fluid bolus, the ideal test for fluid responsiveness would employ maneuvers such as a passive leg raise or, in patients receiving controlled mechanical ventilation, an end-expiratory occlusion test, which do not require fluid administration before determining its need. Portable, handheld ultrasounds may be useful for assessing fluid responsiveness in patients such as those undergoing surgery where it may be impractical to have invasive monitoring (arterial line, pulmonary artery catheter) and other noninvasive measurements (LVOT VTI) due to lack of access to the chest. Furthermore, it is cost-effective as the portable, handheld ultrasound devices can be easily cleaned and immediately reused on another patient without requiring costly, single-use sensors (i.e., bioreactance and esophageal Dopplers). We believe that many of our patients would benefit from portable, handheld ultrasound assessment of ccFT to guide intraoperative fluid administration given its ease of use and the increasing availability and affordability of portable handheld ultrasound devices. Further studies across different patient populations are necessary to validate this technique prior to widespread use.

## Data Availability

Deidentified data is available by written request to the authors.
